# Mechanochemical Preparation, Characterization and Biological Activity of Stable CuS Nanosuspension Capped by Bovine Serum Albumin

**DOI:** 10.3389/fchem.2022.836795

**Published:** 2022-02-15

**Authors:** Martin Stahorský, Zdenka Lukáčová Bujňáková, Erika Dutková, Martin Kello, Bohdan Mahlovanyi, Yaroslav Shpotyuk, Nina Daneu, Jelena Trajić, Matej Baláž

**Affiliations:** ^1^ Department of Mechanochemistry, Institute of Geotechnics, Slovak Academy of Sciences, Košice, Slovakia; ^2^ Faculty of Materials, Metallurgy and Recycling, Technical University of Košice, Košice, Slovakia; ^3^ Department of Pharmacology, Faculty of Medicine, P. J. Safarik University, Košice, Slovakia; ^4^ Institute of Physics, University of Rzeszow, Rzeszów, Poland; ^5^ Department of Sensor and Semiconductor Electronics, Ivan Franko National University of Lviv, Lviv, Ukraine; ^6^ Advanced Materials Department, Jožef Stefan Institute, Ljubljana, Slovenia; ^7^ Institute of Physics, University of Belgrade, Belgrade, Serbia

**Keywords:** copper sulfide (CuS), mechanochemistry, wet stirred media milling, capping, bovine serum albumin (BSA), biological activity

## Abstract

The biocompatible nanosuspension of CuS nanoparticles (NPs) using bovine serum albumin (BSA) as a capping agent was prepared using a two-stage mechanochemical approach. CuS NPs were firstly synthetized by a high-energy planetary ball milling in 15 min by milling elemental precursors. The stability of nanoparticles in the simulated body fluids was studied, revealing zero copper concentration in the leachates, except simulated lung fluid (SLF, 0.015%) and simulated gastric fluid (SGF, 0.078%). Albumin sorption on CuS NPs was studied in static and dynamic modes showing a higher kinetic rate for the dynamic mode. The equilibrium state of adsorption was reached after 90 min with an adsorption capacity of 86 mg/g compared to the static mode when the capacity 59 mg/g was reached after 2 h. Then, a wet stirred media milling in a solution of BSA was introduced to yield the CuS-BSA nanosuspension, being stable for more than 10 months, as confirmed by photon cross-correlation spectroscopy. The fluorescent properties of the nanosuspension were confirmed by photoluminescence spectroscopy, which also showed that tryptophan present in the BSA could be closer to the binding site of CuS than the tyrosine residue. The biological activity was determined by *in vitro* tests on selected cancer and non-tumor cell lines. The results have shown that the CuS-BSA nanosuspension inhibits the metabolic activity of the cells as well as decreases their viability upon photothermal ablation.

## Introduction

Interest in copper sulfide semiconductors has increased since the middle of the 20th century and continues nowadays (Reynolds et al., 1954; Neville, 1995). The most cvommonly studied copper sulfide is covellite, CuS. Its nanocrystals can be used as p-type semiconductors due to the copper vacancies in the lattice framework, which is the reason for their use in optoelectronic devices (Partain et al., 1983; Lou et al., 2003). Its versatility, availability and low toxicity guarantee wide applicability (Feng et al., 2015; Roy and Srivastava, 2015), including the biological field. Namely, copper sulfide nanoparticles (CuS NPs) are suitable for diagnostics, bioimaging, photothermal therapy, or drug delivery (Zhou et al., 2010; Ku et al., 2012; Ramadan et al., 2012; Goel et al., 2014; Liu et al., 2014).

Various methods are used for the synthesis of CuS NPs, namely a solvothermal/hydrothermal (Nørby et al., 2014), colloidal (Han et al., 2008), hot injection (Zhang et al., 2012), or a microwave-assisted method (Nafees et al., 2012). The mechanochemical synthesis using high-energy ball milling method is another option that excels in several aspects (Jones and Eddleston, 2014). It is considered a green method because it is usually performedin a solvent-free mode and does not need a heat supply (Baláž et al., 2013). A further advantage of this method is its versatility, due to the possibility to synthesize organic (James et al., 2012) as well as inorganic materials (Šepelák et al., 2013) with the ability to perform high-temperaturedemanding reactions under laboratory conditions (Ou et al., 2015). In general, mechanochemical synthesis reduces the number of steps and saves time in the preparation of products (Ou et al., 2015). The synthesis of CuS NPs in a laboratory mill in 10 s was described in (Balaz et al., 2016), as well as many other works have reported the successful synthesis of CuS in a short time (Wang et al., 2006; Wang and Tan, 2010; Ou and Li, 2014). On an industrial scale, 60 min of milling was necessary to produce 7,500 g of CuS in one batch using an industrial eccentric vibrating mill (Achimovičová et al., 2019).

In medical applications, nanoparticles work best when they maintain their stability and properties, do not form agglomerates and are non-toxic (Singla et al., 2016). Since the mid-1990s, the surface functionalization of NPs using biomolecules as capping agents has been intensively studied, especially for controlling synthesis, maintaining the stability and properties of NPs. The programmable properties of biomolecules significantly improved the new functions of NPs with the intention of achieving “smart” materials in biodiagnostic, therapeutic, and optical applications (Abou El-Nour et al., 2010; Biju, 2014; Oh et al., 2015).

Bovine serum albumin (BSA) is one of the most commonly used biomolecules for capping of nanoparticles (An and Zhang, 2017). BSA is one of the most abundant and well-characterized proteins present in the plasma of mammals. BSA is composed of 580 amino acid residues. It is a water-soluble and a weak reducing agent. The key role of this protein is to maintain the optimum level of pH in the blood and control the transport of different substances of various natures, such as ionic, hydrophilic and hydrophobic (Carter and Ho, 1994). The use of albumin-capped nanoparticles has been clinically approved several times (An and Zhang, 2017), including sulfide nanoparticles. Namely manganese sulfide for hydrogen sulfide gas therapy and copper sulfide used as a photothermal agent was studied (Chu et al., 2018; Wan et al., 2019; He et al., 2020).

An easy-to-implement way of introducing a biocompatible agent to the inorganic NPs is a wet milling approach. The wet stirred media milling is a frequently applied fully scalable top-down method towards the production of micron- and nanoscale suspensions (Afolabi et al., 2014; Romeis et al., 2016; Bilgili and Guner, 2021). The main difference compared to dry milling is the introduction of a liquid dispersion medium. The introduction of this parameter as well as the mechanical deformation in the solid structure increase the reactivity of the substance. During this type of milling, not only interactions between solids, milling balls and chamber take place, but also water (solvent), comes into play. The overall effect of water is controlled by the nature of the solid (porosity, crystallinity, polarity, etc.), the genesis and amount of water, the type of mill, the milling mechanism and the milling conditions (milling time, milling intensity, etc.) (Baláž et al., 2013; Romeis et al., 2016). Because chemical reactions of stressed particles with the liquid phase can also occur, some materials (soluble in water) cannot be milled in the wet conditions. Thus, water-insoluble NPs and the use steric, electrostatic or electro-steric stabilizers, are required to form a stable nanosuspension to form a stable nanosuspension (Mucsi, 2019). The advantage of wet milling is its high energy density generated in the milling chamber (3–10 times higher than in other high-energy mills (HEM)) (Mucsi, 2019). Furthermore, the ability of continuous operation, the high number of variable parameters (circumferential speed, size of balls, material, wet or dry state, drying in a mill) make it a well-controllable method of nanosuspension production (Delogu and Mulas, 2010; Romeis et al., 2016; Mucsi, 2019; Parker et al., 2020). However, discussions are still ongoing to provide a comprehensive understanding of the impact of different parameters on product properties (Baláž et al., 2013; Afolabi et al., 2014; Romeis et al., 2016; Fragnière et al., 2018; Mucsi, 2019; Parker et al., 2020; Bilgili and Guner, 2021). The wet milling method has been used several times for mechanical activation, nanoscale product formation, as well as nanosuspension preparation (Stenger et al., 2005; Niwa et al., 2011; Jung et al., 2015; Kuroiwa et al., 2018; Karuppannan et al., 2019). Using this technique, our research group was able to prepare stable nanosuspensions with interesting magnetic, optical and therapeutic properties by combining inorganic materials with bioactive and biocompatible capping agents in the past (Bujňáková et al., 2017a; Bujňáková et al., 2017b; Bujňáková et al., 2020; Dutková et al., 2020).

In this paper, the biocompatible CuS nanosuspension capped by BSA was prepared in an eco-friendly way using a two-step mechanochemical approach for the first time. In the first step, the CuS NPs were prepared using a planetary ball mill (according to (Baláž et al., 2017)) and in the second step, the wet stirred media milling in the presence of BSA was performed. The CuS NPs were studied in more detail in terms of stability in simulated human body conditions. Subsequently, the adsorption capacity of BSA on these nanoparticles was studied. Biofunctionalization of nanocrystals in an aqueous BSA solution was performed using a circulating ball mill (MiniCer). The prepared nanosuspension was studied in terms of its structure, long-term stability and fluorescent properties. In addition, *in vitro* experiments showed the concentration-dependent ability of CuS-BSA nanocrystals to inhibit metabolic activity on all studied cell lines, both tumor and non-tumor. The nanocrystals showed an efficiency to kill tumor cells upon irradiation in the NIR region. Finally, the study deals with the optimization of nanocrystals concentration and irradiation time, which are essential for the safety of CuS-BSA nanocrystals in biomedical applications.

## Materials and Methods

### Materials

Electrolytically prepared copper (99+%, Merck, Germany), sulfur (99%, CG-Chemikalien, GmbH, Germany), bovine serum albumin (BSA, for biochemistry, protease free, Acros Organics), buffer aqueous solution (phosphate buffered saline tablets, Fisher BioReagents, pH = 7.4), NaCl, NaOH, HCl, Na_2_HPO_4_ (all from ITES, Slovakia), KCl, CaCl_2_.2H_2_O, KH_2_PO_4_, Na_2_HPO_4_.H_2_O, MgSO_4_.7H_2_O, MgCl_2_.6H_2_O (all from Lachema, Czech Republic), Na_2_SO_4_, CH_3_COONa, NaHCO_3_, sodium citrate dihydrate (all from Centralchem, Slovakia), dodecyl sulfate sodium salt (Merck, Germany), pepsin and dextrose (all from Fischer Chemical, United Kingdom) were used without further purification in the syntheses and preparation of simulated body fluids (SBF).

### Mechanochemical Synthesis

CuS NPs were prepared by mechanochemical synthesis using a planetary ball mill (Pulverisette 7 Premium line, Fritsch) in a special tungsten carbide milling chamber with the volume of 80 ml, as described in more details in (Baláž et al., 2017). Overal mass of three grams containing Cu (1.994 g) and S (1.006 g) was milled employing following conditions: atmosphere – air, 18 tungsten carbide milling balls with the diameter 10 mm, ball-to-powder ratio 47, rotation speed of the planet carrier– 500 rpm, milling time – 15 min. The milling was repeated more times to obtain enough material for subsequent wet stirred media milling process.

**FIGURE 1:**
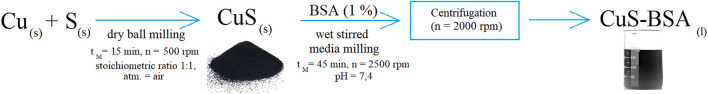
The CuS-BSA nanosuspension preparation scheme.

The preparation of the CuS-BSA nanosuspension was performed by the wet stirred media milling method using 5 g of CuS NPs (prepared according to the procedure described above) and 300 ml of BSA aqueous buffer solution (1% wt., PBS tablets for pH = 7.4), in a MiniCer circulating mill (Netzsch, Germany). The milling conditions were as follows: milling time 45 min, the milling speed of 2,500 rpm, 140 ml of yttrium-stabilized ZrO_2_ balls (0.6 mm in diameter). After the milling, the samples were centrifuged at 2000 rpm. Afterwards, the nanosuspension was characterized and stored in a refrigerator (at 4°C). The reproducibility of the synthesis was verified by repeating the CuS-BSA nanosuspension preparation step more times. Under the same conditions (*see* Electronic Supplementary File, Supplementary Figure S1). The scheme of preparation of CuS capped by BSA is described in Figure 1.

### Characterization Methods

To determine the stability of the prepared CuS NPs, the solubility tests were performed by leaching prepared CuS NPs (100 mg) at simulated human body temperature (36.5°C) for 120 min in 100 ml of demineralized water (H_2_O), physiological solution (0.9% NaCl) and five simulated body fluids (SBF): *1*) PBS (phosphate buffered saline, simulated human extracellular fluid), *2*) SGF (simulated gastric fluid), *3*) SIF (simulated intestinal fluid), *4*) Hank’s saline (simulated cell culture), and *5*) SLF (simulated lung fluid). After leaching, the samples were filtered and the filtrate was collected for analytical quantification of copper by atomic absorption spectrometry - SPECTRAA L40/FS (Varian, Australia).

The adsorption of BSA from an aqueous buffer solution (phosphate buffered saline tablets, Fisher BioReagents, pH = 7.4) on the surface of CuS NPs was studied with 1% BSA solution (10 mg/ml) at different adsorption times (1–120 min) in static (no stirring) as well as dynamic mode (samples were shaken on a laboratory shaker): m(CuS) = 0.2 g, V(BSA) = 10 ml, initial pH of the solution ∼7.4, T = 25°C. After determining the equilibrium adsorption time (90 min), the experiments were performed with different concentrations of BSA (0.1–1%). After the adsorption, the samples were filtered and the filtrate was collected to record UV-Vis spectra.

Zeta potential (ZP) was measured using a Zetasizer Nano ZS (Malvern, United Kingdom). The zetasizer measures the electrophoretic mobility of the particles, which is converted to the ZP using the Smoluchowski equation built into the Malvern zetasizer software. All measurements were performed in the pH range from 3 to 11/12. The measurements were repeated three times for each sample.

The particle size distribution was measured by PCCS (photon cross-correlation spectroscopy) using a Nanophox particle size analyzer (Sympatec, Germany). A portion of the nanosuspension was diluted with the solution of BSA to achieve the appropriate concentration for measurement. This analysis was performed using a refractive index of dispersant of 1.33. The measurements were repeated three times for each sample.

The following samples were used for further characterization of the nanosuspension: *1*) the original nanosuspension, *2*) the dried sample (nanosuspension dried at room temperature (r.t.)) and *3*) the washed sample (the dried sample washed with distilled water). In the last case, 0.5 g of dried sample was washed with 50 ml water using vigorous shaking on a laboratory shaker and dried again at r.t.

Microstructural characterization of the original nanosuspension was performed by a transmission electron microscopy (TEM) using a 200-kV microscope JEM 2100 (Jeol, Japan) with LaB_6_ electron source Si(Li). A droplet of the nanosuspension was applied onto the carbon-coated Ni grid and dried. Prior to the TEM analyses, the sample was additionally coated with a thin layer of carbon in order to prevent charging of the sample and under the electron beam. The microstructure and morphology of the washed sample were also studied using the TEM FEI Tecnai Osiris device with a primary electron beam accelerated by 200 kV voltage. A chemical composition mapping was performed by EDS.

XRD patterns of studied CuS NPs were collected at room temperature in the angular range 10° < 2*θ* < 100°, using Bruker D8 Advance (with Cu anode λ = 1.5406 Å as X-ray source).

Fourier transform infrared (FT-IR) spectroscopy was performed using a Tensor 27 spectrometer (Bruker, Germany) using the ATR method. The samples were measured in the frequency range of 4,000–650 cm^−1^. The spectra were expressed as absorbance versus wavenumber (cm^−1^).

Raman spectra were recorded with a Renishaw inVia Raman Microscope. Argon laser Stellar-REN with a wavelength of 488 nm and power of 50 mW (10% of the power was set during measurements) and semiconductive laser with a wavelength of 785 nm and power of 200 mW (10%) were used as a source of excitation. Additional settings: exposure time - 10.00 s, accumulations -2, objective- ×50. The measurements were performed in reflection mode at room temperature.

UV–Vis spectroscopy was performed for samples subjected to adsorption kinetics study and for the final CuS-BSA nanosuspension. The absorption UV–Vis spectra were recorded using a UV–Vis spectrophotometer Helios Gamma (Thermo Electron Corporation, Great Britain) in the range 200–800 nm using a 1 cm light quartz cuvette.

Optical properties were also studied by photoluminescence (PL) spectroscopy. The fluorescence quenching spectra at room temperature were acquired at a right angle on a photoncounting spectrofluorometer PC1 (ISS, United States) with an excitation wavelength of 280 nm. A 300 W xenon lamp was used as the excitation source. Excitation and emission slit widths were set to 0.5 and 1 mm, respectively. One-cm-path length rectangular quartz cuvette was used for the measurement. The intervals between maximum excitation and emission wavelength were set as Δλ = 15 nm for tyrosine and Δλ = 60 nm for tryptophan residues.

The effect of CuS-BSA nanosuspension on cell viability and anticancer activity was studied *in vitro*. Human colorectal carcinoma (HCT116), and human cervical adenocarcinoma (HeLa) cell lines were cultured in RPMI 1640 medium (Biosera, Kansas City, MO, United States). The human breast carcinoma (MDA-MB 231), human colorectal adenocarcinoma (CaCo-2), human lung carcinoma (A549), human breast adenocarcinoma (MCF-7), human epithelial kidney (HEK293) and human kidney fibroblast (COS-7) cell lines were maintained in growth medium consisting of high glucose Dulbecco’s Modified Eagle Medium with sodium pyruvate (GE Healthcare, Piscataway, NJ, United States). All media were supplemented with a 10% fetal bovine serum (FBS), penicillin (100 IU/ml) and streptomycin (100 μg/ml) (all Invitrogen, Carlsbad, CA, United States) in an atmosphere containing 5% CO_2_ in humidified air at 37°C. Cell viability, estimated by trypan blue exclusion, was greater than 95% before each experiment. The metabolic activity colorimetric assay (MTS) was used to determine effects of BSA alone (c = 0.75–100 μg/ml) and CuS-BSA nanosuspension (c(Cu) = 0.75–100 μg/ml) on the metabolic activity of different cell lines. After 72 h of incubation, 10 μl of MTS (Promega, Madison, WI, United States) were added to each well according to the CellTiter 96^®^ Aqueous One Solution Cell Proliferation Assay protocol. After a minimum 1 h incubation, the absorbance was measured at 490 nm using the automated Cytation™ three Cell Imaging Multi-Mode Reader (Biotek, Winooski, VT, United States). The absorbance of the control wells was taken as 100% and the results were expressed as a percentage of the control. All experiments were performed in triplicate and data were used to IC50 calculation.

For estimated flow cytometry analysis of cell granularity and fluorescence microscopy analysis, the cells (HCT116, HeLa and MDA-MB-231) were seeded at a density of 3 × 10^4^ in Petri dishes (Sarstedt, Nümbrecht, Germany). Twenty-four hours after cell seeding, the cells were treated with the CuS-BSA NPs (c = 0.7–100 μg/ml) for 72 h, washed two times with 1X PBS (Sigma-Aldrich) and harvested by trypsinization. Uptake of nanoparticles to the different cell lines was analyzed through granularity (SSC-H vs FSC-H) and fluorescence (FL-1, FL-2, FL-3) changes on FACSCalibur flow cytometer and FlowJo software (Becton Dickinson, San Jose, CA, United States).

Photothermal ablation was also studied on HCT116, HeLa and MDA-MB-231 cell lines. The samples were irradiated using BEURER IL50 irradiator, 300 W, Germany from a distance of 30 cm for 5, 10 and 20 min with analysis after 48 h was performed. The energy of lamp is 0.049 W/cm^2^ in a distance of 30 cm from the source.

Results were expressed as mean ± SD. For statistical analysis, the one-way ANOVA followed by the Bonferroni multiple comparisons test was used. Differences were considered significant when *p* < 0.05.

## Results and Discussion

### The NPs Stability in the Presence of Simulated Body Fluids (SBF)

In the first step, the CuS the leachability of copper from CuS NPs into simulated body fluids was investigated. Quantitative determination of copper leaching of the CuS NPs in most simulated body fluids revealed zero copper concentration in the leachates, except simulated lung fluid (SLF, 0.015%) and simulated gastric fluid (SGF, 0.078%). These measured values are influenced by the acid-base properties of the simulated body fluids (SLF pH 8.7, SGF pH 1.1 and neutral pH of H_2_O, NaCl 0.9%, PBS, SIF, Hank’s saline) and higher concentrations of ionic substances in SLF and SGF. SLF is basic, whereas SGF is acidic, and under both conditions, copper is better dissolved. In general, the results showed zero/very low solubility of the CuS NPs in simulated human body conditions. The demonstrated stability of nanoparticles forms the basis for their potential medical use.

**FIGURE 2:**
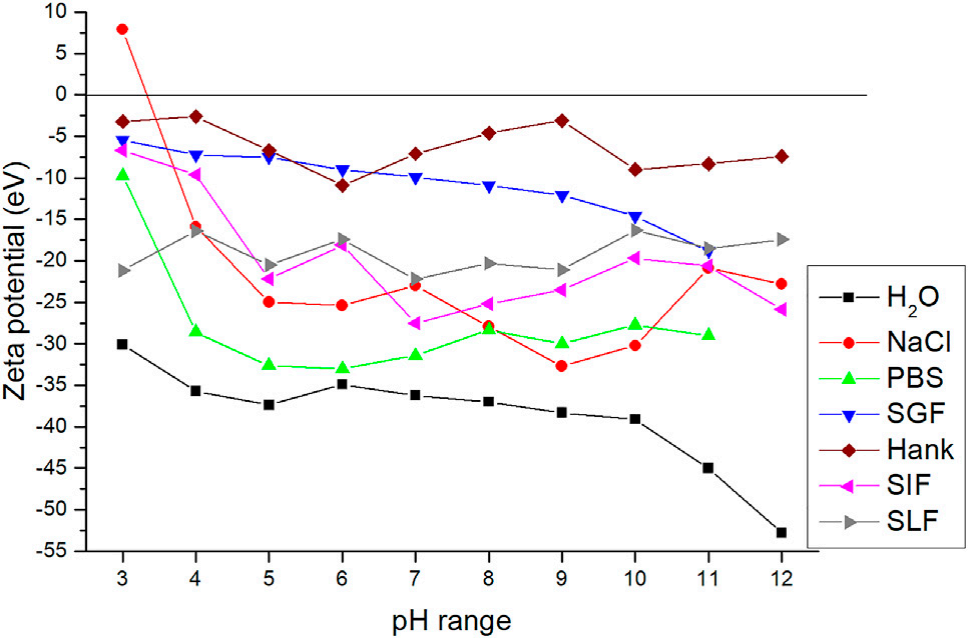
Dependence of zeta potential on pH environment for the CuS NPs in SBF.

To have a closer look on the CuS stability, zeta potential measurements were performed. The CuS samples showed mostly negative zeta potential values, being the most negative when they were dispersed in demineralized water (black curve, Figure 2). When the ionic strength in fluid solutions increased, the ZP values moved to the less negative values, up to almost zero for Hank’s saline. Only in the case of 0.9% NaCl solution positive ZP value was observed with the isoelectric point (IEP) at pH 3.33.The literature states that the initial surface charge can be compensated much faster, if more ions are available, leading to lower ZP in absolute terms. With decreasing electrolyte concentration, the ZP value increases in either a negative or positive direction, depending on the nature of the electrical bilayer of nanoparticles (Carneiro-Da-Cunha et al., 2011). Negative CuS values are characteristic of unoxidized sulfides and are consistent with the literature (Liu and Huang, 1992).

### Preparation and Characterization of the CuS-BSA Nanosuspension

#### Sorption Kinetics and Isotherm

**FIGURE 3:**
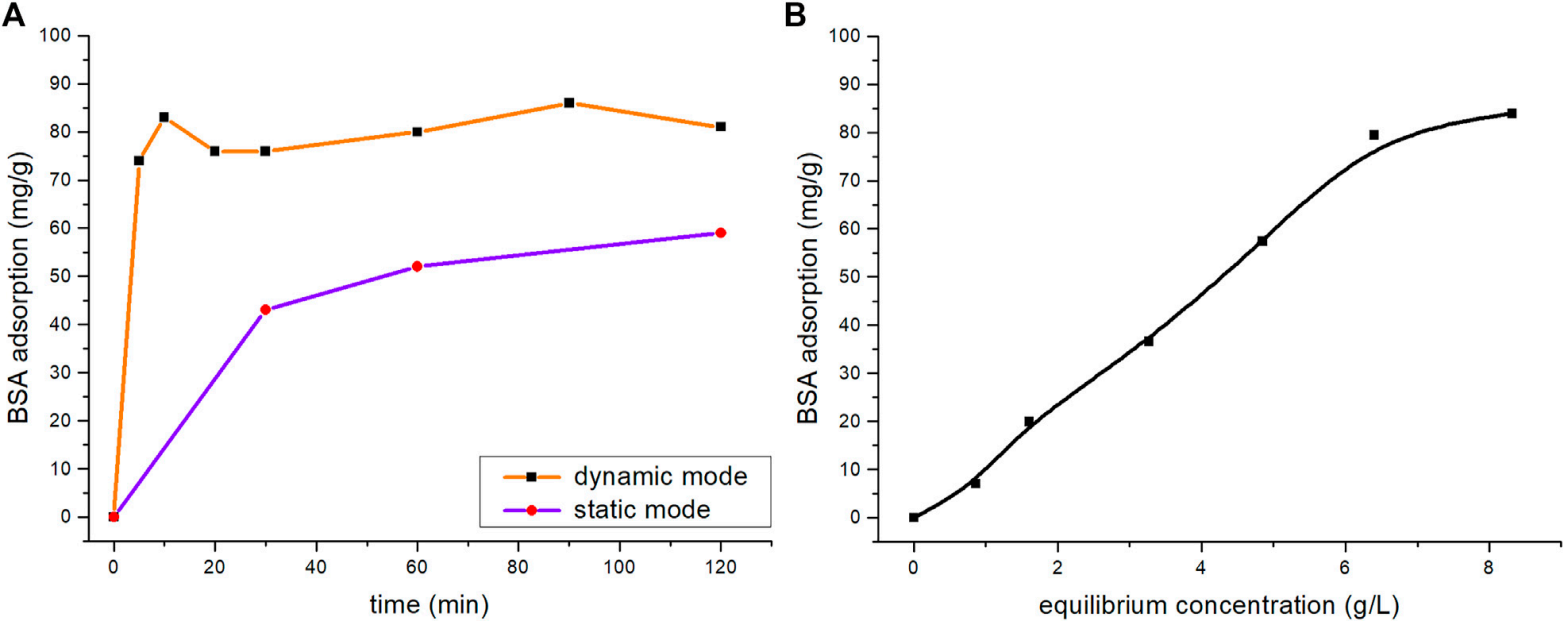
Sorption kinetics of the BSA adsorption on CuS NPs for: **(A)** dynamic and static modes and **(B)** sorption equilibrium isotherm.

For successful capping of the CuS NPs with albumin, an interaction between these entities *via* e.g. adsorption should exist. In order to determine whether BSA adsorption on the surface of the CuS NPs is possible, the adsorption kinetics and adsorption capacity in the dynamic and static mode were studied (Figure 3A). As the adsorption curves suggest, the kinetic rate was higher for the dynamic mode. In the dynamic mode, the equilibrium state of adsorption was reached after 90 min with an adsorption capacity of 86 mg/g. The amount of adsorbed BSA in the static mode reached 59 mg/g after 2 h.

The dynamic mode at 90 min was chosen for further study and the sorption isotherm was constructed expressing the relationship of BSA distribution between the liquid and solid phases at different concentrations (Figure 3B). The amount of the adsorbed BSA increased in the initial isotherm and reached a plateau at 84 mg/g.

#### Preparation of CuS-BSA Nanosuspension by Wet Stirred Media Milling

**FIGURE 4:**
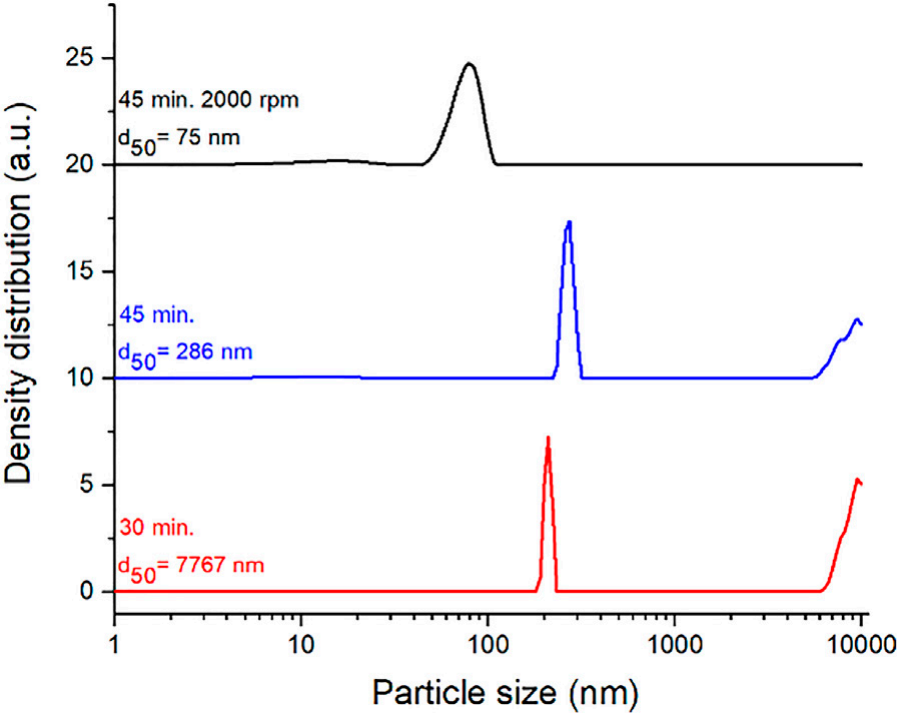
Progress of particle size distribution during wet stirred media milling of CuS in BSA solution. Milling time, revolutions of centrifugation and average hydrodynamic diameter (d_50_) are defined in the figure.

To obtain well-dispersed colloidal CuS nanocrystals suitable for testing of biological activity and bio-imaging properties in cancer cells, the wet stirred media milling process using BSA was successfully applied. The change of particle size distribution during the processing (after 30 and 45 min of milling) is summarized in Figure 4. The additional centrifugation at 2,000 rpm was required to obtain the sample with unimodal particle size distribution with the average hydrodynamic diameter (d_50_) 75 nm. This result is in accordance with the one from TEM analysis (described later). Compared to other systems prepared by the wet milling method in the laboratory circulation mill (MiniCer), these are less demanding conditions. Parameters such as total preparation time, milling speed as well as centrifugation speed were reduced individually by tens of percentin comparison with the conditions used for other systems (Bujňáková et al., 2017a; Bujňáková et al., 2020; Dutková et al., 2020).

**FIGURE 5:**
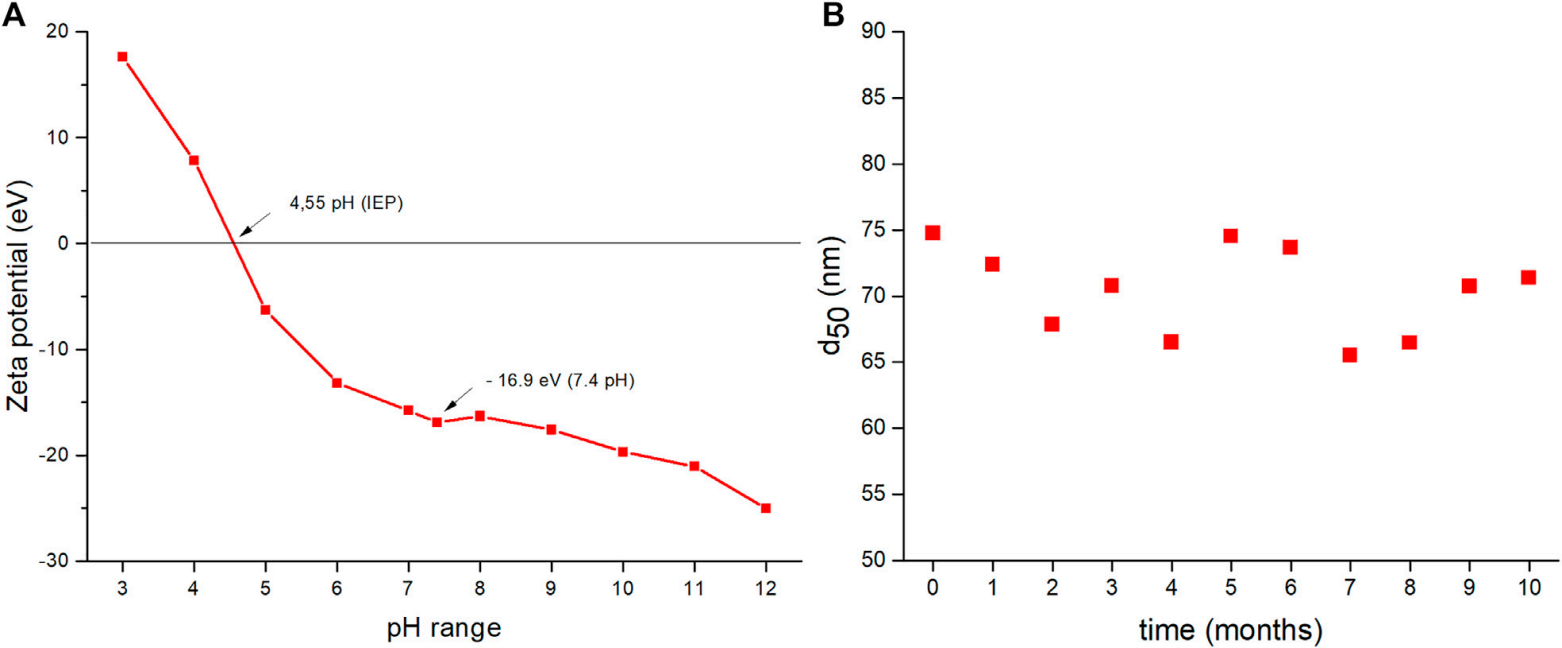
**(A)** Dependence of zeta potential on pH, **(B)** long-term stability of BSA-capped CuS (dependence of d_50_ value on time of storage).

In terms of stability, agglomeration could occur in nanosuspension in general, which can result in faster activation of processes that cause colloidal system instability as a result of Ostwald ripening (Boistelle and Astier, 1988). Therefore, ZP measurements and long-term stability studies were used for this system. ZP was measured as a function of the applied pH (in a range from 3 to 12) in the original BSA-containing dispersions obtained after milling. The results are shown in Figure 5A. As the pH increases, the detected ZP values move from positive to negative values, which replicates the curves for the CuS NPs in other simulated body fluids presented in Figure 2. The isoelectric point was observed at pH 4.55. At the natural pH of the nanosuspension (7.4), the ZP value was −16.9 eV. The particle size distribution was monitored monthly (up to 10 months) in order to determine the long-term stability (Figure 5B). The unimodal distribution of the CuS-BSA sample did not change with time until 10 months (the average D_50_ value was 70.42 nm ± 4.9), indicating very good stability.

#### TEM Analysis of CuS-BSA Nanosuspension

**FIGURE 6:**
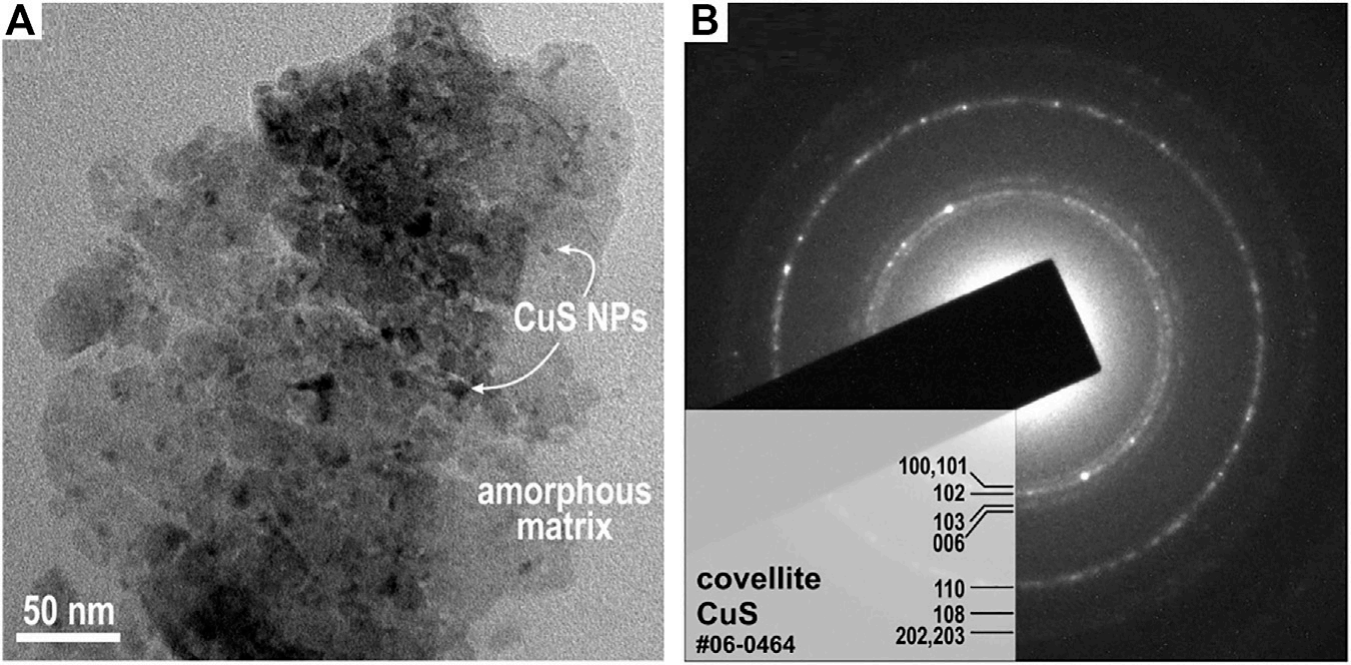
**(A)** Typical TEM image of the CuS-BSA nanosuspension shows CuS NPs embedded in the amorphous organic matrix. **(B)** SAED pattern of the CuS-BSA nanosuspension.

To confirm the successful capping of CuS NPs, TEM analysis was performed on the CuS-BSA nanosuspension sample (Figure 6). The TEM image in Figure 6A reveals that the sample is composed of agglomerates in which nanocrystalline particles with size below 20 nm are surrounded by amorphous organic matrix, most probably the albumin corona (Peng et al., 2013). Selected area electron diffraction (SAED) pattern recorded from the agglomerated particle is shown in Figure 6B. The d-values of the diffraction rings confirm that the nanocrystalline particles are the covellite, CuS (JCPDS 04-0464) phase.

#### Optical Properties of CuS-BSA Nanosuspension

**FIGURE 7:**
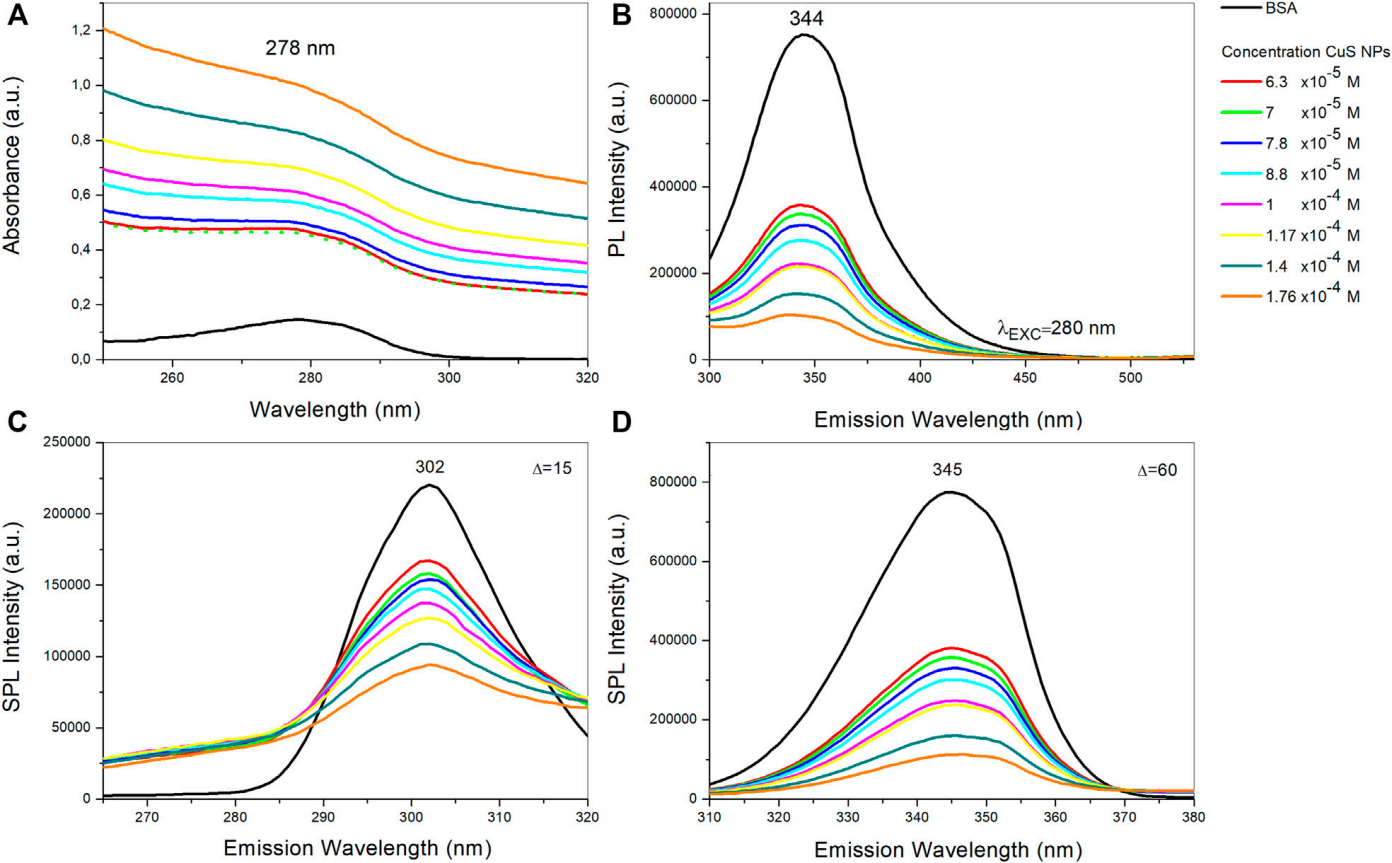
**(A)** UV-Vis absorption spectra, **(B)** fluorescence emission spectra, **(C)** synchronous fluorescence spectra at Δλ = 15 nm, and **(D)** at Δλ = 60 nm of BSA and BSA with various concentrations of CuS NPs.

The optical properties of the CuS-BSA nanosuspension in a concentration-dependent setup were studied by UV-Vis (Figure 7A) and PL spectroscopy (Figures 7B–D). The UV–Vis spectrum of the nanosuspension showed a characteristic absorption peak at 278 nm, revealing the presence of albumin due to the transition of π-π* amino acid residues, such as tryptophan, tyrosine or phenylalanine (Zhao et al., 2010). As the concentration of CuS NPs increases compared to pure BSA, the absorption of the CuS-BSA system also increases, but its absorption maximum does not shift. It could mean that the microenvironment around the amino acid residues in BSA does not change during complex formation between BSA and nanoparticles (Li et al., 2016; Prasanth et al., 2018).

Due to the presence of tryptophan, tyrosine and phenylalanine residues, BSA has intrinsic fluorescence. In particular, the tryptophan residue is most sensitive to tiny changes in the microenvironment and becomes a profound indicator of changes in the secondary conformation. The pure BSA aqueous solution at room temperature exhibits two strong intrinsic fluorescence emission bands centered at 345 and 292 nm, respectively. The BSA band at 345 nm originates from the emission of tryptophan residues, and the band at 292 nm is ascribed to the emission of tyrosine residues. Thus, fluorophore quenching and the resulting reduction in fluorescence emission is a useful indicator of protein binding (Wu et al., 2011; Chen and Wu, 2012; Prasanth et al., 2018).

The fluorescence quenching spectra of BSA and CuS-BSA with different concentrations of CuS NPs were measured after excitation at 280 nm (Figure 7B). In our case, the sensitive emission band for tryptophan residues in BSA was detected at 344 nm. Fluorescence quenching is present in any process that reduces the fluorescence intensity of the sample. The fluorescence intensity of CuS-BSA samples is several times lower compared to pure BSA, and at the same time it further decreases with increasing concentrations of the CuS NPs (opposite to absorbance). This suggests that the chromophore residues of the protein (BSA) are repressed by CuS NPs. The CuS NPs interact with BSA by generating the non-fluorescence complex between them – so-called ground state complex. Since there is no peak shift during quenching, changes in the microenvironment near tryptophan residues are unlikely (Khani et al., 2011; Li et al., 2016; Prasanth et al., 2018).

Synchronous fluorescence spectroscopy is used to study the molecular environment. It can provide information on the proximity of amino acid residues, such as tryptophan and tyrosine in BSA, to the CuS NPs. The values of the difference between emission and excitation wavelengths (Δλ) are determined for these amino acid residues (Miller, 1979), for tyrosine Δλ is 15 nm, and for tryptophan Δλ is 60 nm. These values provide information about changes in their microenvironment. Increasing the concentration of CuS NPs leads to a significant decrease in fluorescence intensity. The decrease in fluorescence intensity for tyrosine residues (Figure 7C) was 57.2%, whereas the decrease in fluorescence intensity for tryptophan (Figure 7D) was 85.5%. Fluorescence from tryptophan is about 28% more intense and is more effectively attenuated than fluorescence from tyrosine residues in the presence of CuS, suggesting that tryptophan may be closer to the binding site than tyrosine (Li et al., 2016).

### Characterization of the CuS-BSA Powder

As most of the relevant characterization techniques are relevant for powder samples, we subjected our produced nanosuspension to drying at laboratory temperature. However, as will be demonstrated below, it turned out that there is a significant amount of NaCl in this sample. Thus, samples were further washed with distilled water and dried again to yield “washed” powder. These two samples are compared using X-ray diffraction and FTIR spectroscopy.

#### Comparison of Dried and Washed Nanosuspensions

**FIGURE 8:**
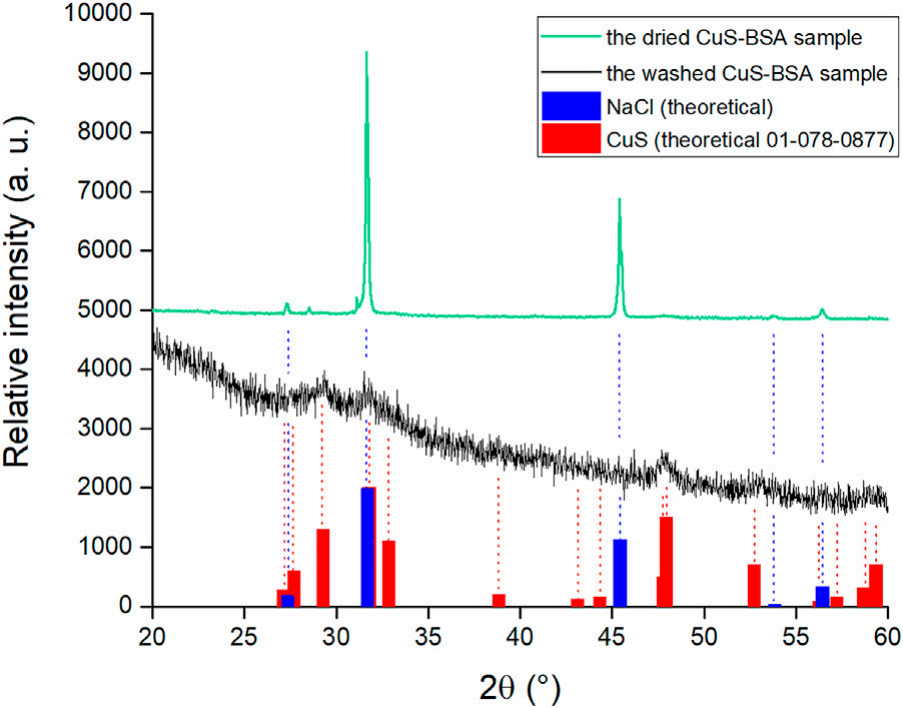
The XRD patterns of the dried nanosuspension and the washed powder.

To clearly identify the crystalline components in the sample, the dried nanosuspension was analyzed by X-ray diffraction. The XRD pattern of the dried sample before washing (green color, Figure 8) showed two major peaks that were assigned to NaCl (cubic centered face, spatial group Fm-3m) using a PDF2 database. The large prevalence of NaCl is due to the use of phosphate buffered saline (PBS) containing sodium chloride as the main component. PBS is used to ensure a neutral pH during milling. After the milling procedure, we have performed centrifugation and only worked with the supernatant then. During centrifugation, a considerable amount of coarser CuS particles is lost in solid, however, NaCl is in the dissolved form in the solution and thus stays in the supernatant. As a result, very intensive peaks of NaCl appear in the XRD pattern after drying of the nanosuspension due to a considerable NaCl crystallization during drying. The XRD pattern of the washed CuS-BSA sample (black color, Figure 8) was XRD-amorphous, however, very broad peaks corresponding to the CuS phase (PDF2 01-078-0877) could be identified. The peaks of NaCl were absent, thus confirming successful washing out.

**FIGURE 9:**
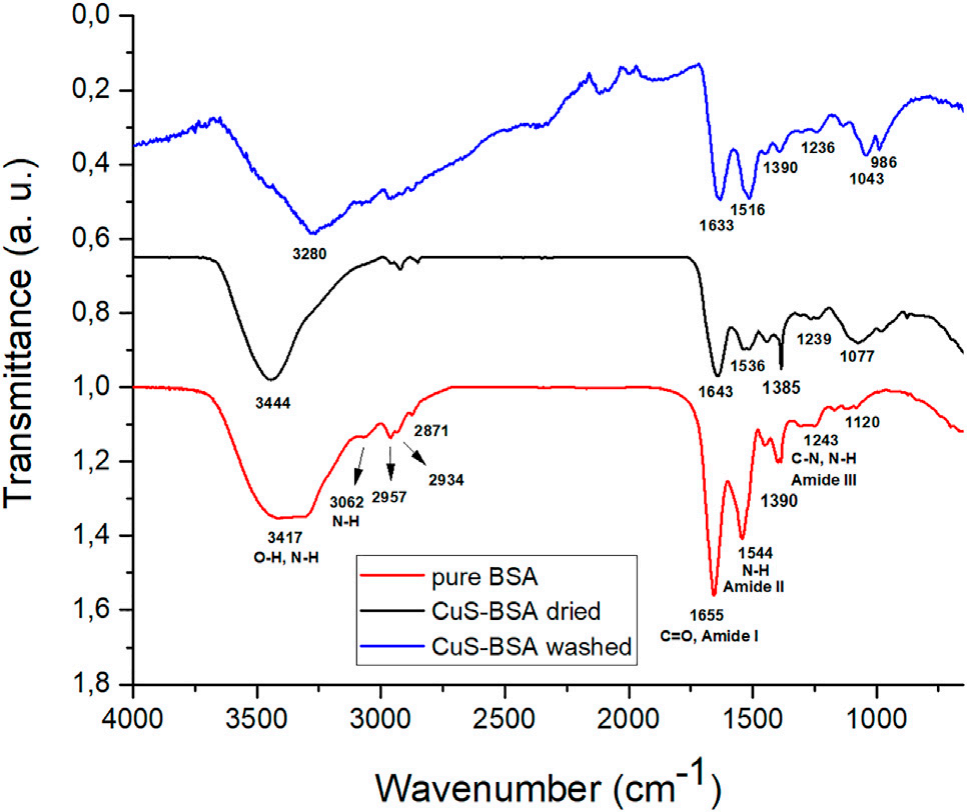
FT-IR spectra: pure BSA (red curve), the dried CuS-BSA sample (black curve) and the washed CuS-BSA sample (blue curve).

To investigate the role of various functional groups of BSA in the capping of CuS NPs, the FTIR spectra for pure BSA, the dried and washed nanosuspension sample were recorded (Figure 9). By comparing FTIR spectra, it is possible to examine changes in the secondary structure of BSA, which are reflected in changes in intensity and shifts of characteristic bands. The major absorption peaks of pure BSA are at 3,417, 3,062, 1,655 and 1,544 cm^−1^. The most intense absorption band at 3,417 cm^−1^ is assigned to –NH stretching vibration, which overlaps with the vibration of –OH from hydroxyl group. Low-intensity absorption bands in the range of 2,957–2,871 cm^−1^ are characteristic of symmetrical and asymmetric alkane chains in the protein. The most sensitive absorption band for changes in the secondary structures of proteins is located at 1,655 cm^−1^ and it corresponds to the carbonyl C=O stretching vibration of amide I band. The absorption band of amide II at 1,544 cm^−1^ belongs to NH-bending vibrations and the absorption band at 1,243 cm^−1^ refers to amide III band, which is caused by C-N and N-H vibrations (Lazarevic et al., 2009; Huang et al., 2010; Shi et al., 2012).

The significant shift in the amide band positions of BSA-adsorbed samples indicates a strong interaction of BSA with the CuS surface atoms. This shift may also be influenced by the conformational change of BSA after the interaction (Dasgupta et al., 2010). In general, a reduction in the intensity of amide II band is considered as a consequence of protein unfolding upon surface interaction (Hartmann, 2005). Changes in positions were observed in the vibration stretching range for hydroxyl groups (3,200–3,500 cm^−1^). Here, also a difference in the spectra of washed and dried nanosuspensions was observed, namely a peak at 3,444 cm^−1^ is visible for the dried sample and a peak at 3,280 cm^−1^ for the washed sample. This difference indicates a difference in chemistry of the two samples (Huang et al., 2010; Bonnier et al., 2017), namely the NaCl which was present in the dried nanosuspension could have some interactions with BSA functional groups, but after its removal, these have vanished.

#### Characterization of the Washed Powder

As it was found that the powder obtained after drying the nanosuspension and subsequent washing to remove NaCl was more relevant to describe the output of the experiment, this was selected for analysis by more characterization techniques.

**FIGURE 10:**
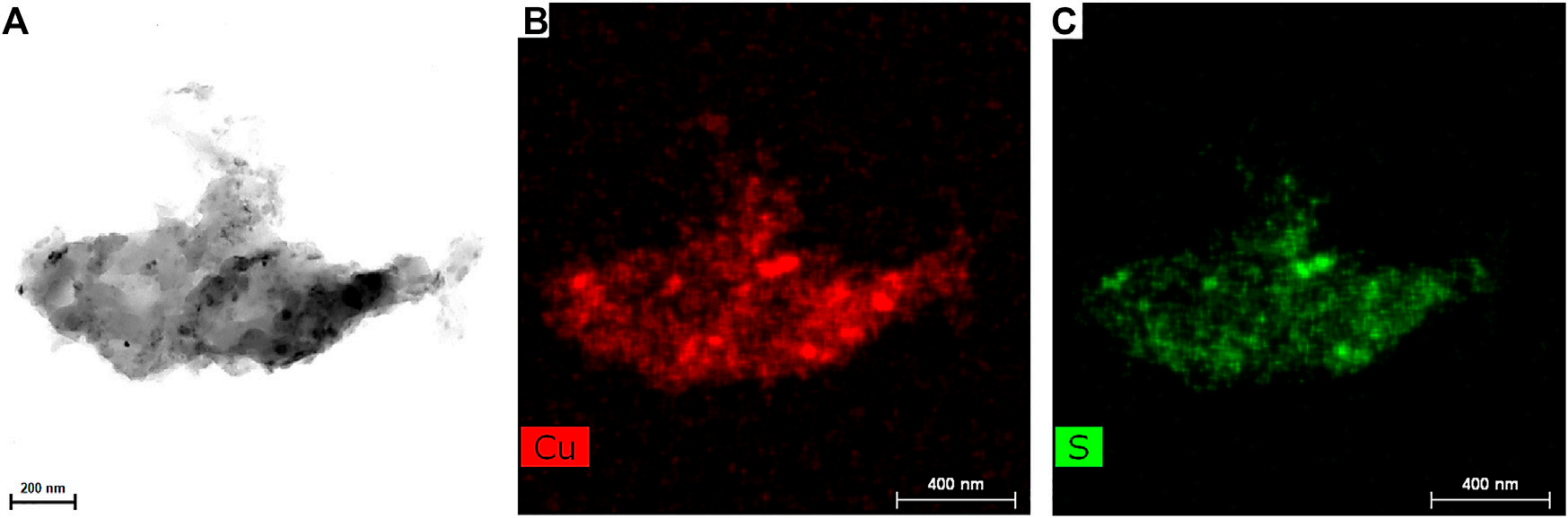
**(A)** The high-angle annular bright-field TEM image, **(B**,**C)** EDS images for elemental mapping Cu and S elements of the washed CuS-BSA sample.

TEM analysis of the washed powder can be found in Figures 10A–C. The TEM image in Figure 10A shows an agglomerate of almost a half micron in size. However, it is clear that it is composed of smaller NPs with a size below 20 nm coated with the albumin corona. The CuS NPs size detected for the washed sample is in accordance with that observed for the nanosupension in Figure 6. EDS mapping of the sample showed the presence of Cu and S elements, perfectly matching the locations of each other, this being clear that CuS compound is present.

Hexagonal CuS crystals have a space group D_6h_^4^ and a primitive unit cell which contains twelve atoms, six of Cu, and six of S. Group theory analysis predicts eight Raman active modes of the zone-center for this crystal: 2A_1g_+2E_1g_+4E_2g_ (Hurma and Kose, 2016).

**FIGURE 11:**
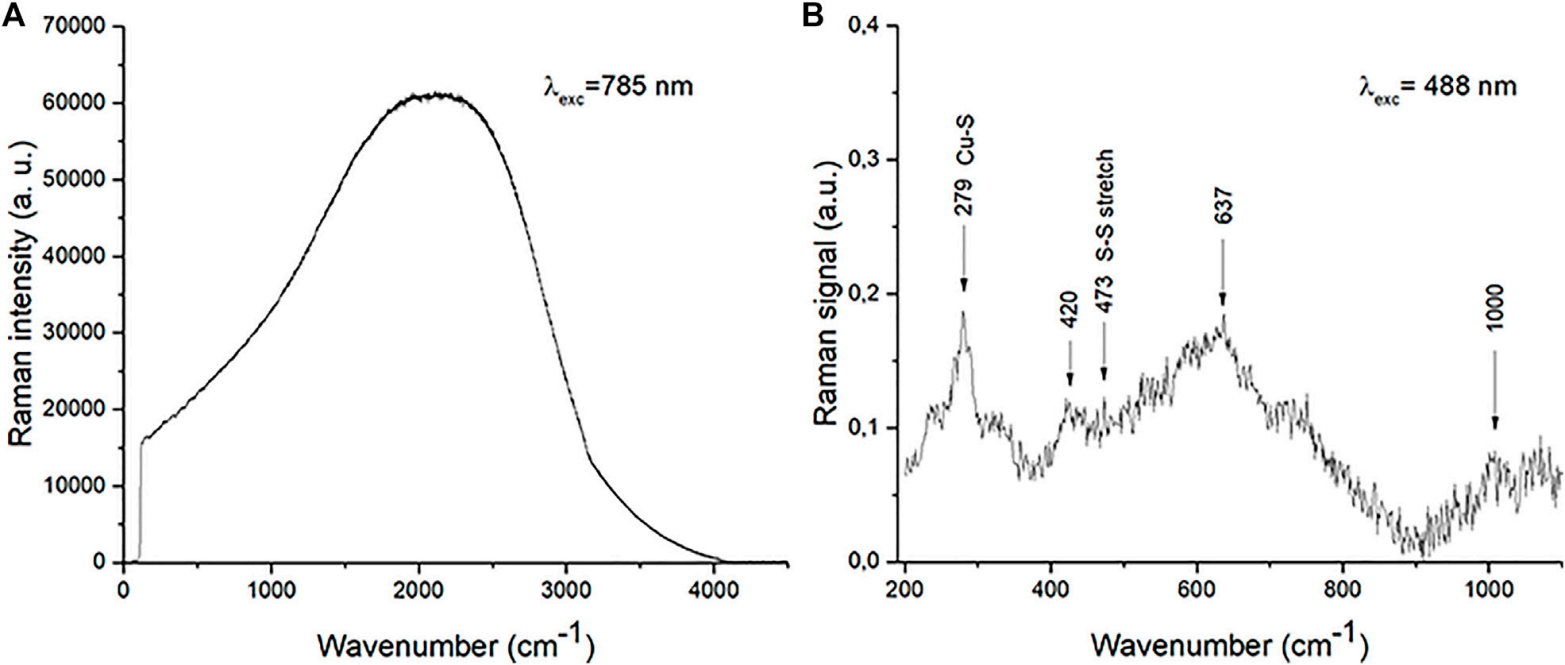
Raman spectra of the washed CuS-BSA sample **(A)** excited under 785 nm wavelength and **(B)** excited under 488 nm wavelength with background subtraction.

In Figure 11A Raman spectra obtained using a laser source of 785 nm is presented. Since the energy of the incident laser light is 1.58 eV which is very close to the CuS band gap (1.55 eV) resonance takes place and indicates that CuS is formed.

Raman spectra obtained using a laser source of 488 nm is presented in Figure 11B. Energy of the incident laser light is 2.54 eV hence among lattice vibrations the occurrences connected with electron transitions are clearly visible. Namely, structures above 1,000 cm^−1^ present electron transitions between states which are mainly derived from Cu 3d states and some S 3p states (Xiao et al., 2016). Structures that originate from lattice vibrations are situated bellow these frequencies. We registered modes at about 279, 420, 473 and 637 cm^−1^. The mode around 473 cm^−1^ is identified as the S-S stretching mode of S_2_ ions at the 4e sites (Hurma and Kose, 2016; Jadhav and Bhuse, 2019). The modes at 473 cm^−1^ (A_1_ (LO) mode) and 264 cm^−1^ (A_1_ (TO) mode) are typically observed modes in Raman spectra of CuS (Brus et al., 2016; Hurma and Kose, 2016). The vibrational mode at 279 cm^−1^ is located between 264 and 290 cm^−1^ CuS mode frequencies, which indicates that it is a combination of the two. Modes registered at 420 and 637 cm^−1^ originated from copper oxide (Cakir, 2017). Copper oxide is not registered in XRD patterns, which indicates that its amount is very small. The shifting of obtained modes compared to bulk mode frequencies is a consequence of the miniaturization.

### Biological Activity: *In Vitro* Studies

**FIGURE 12:**
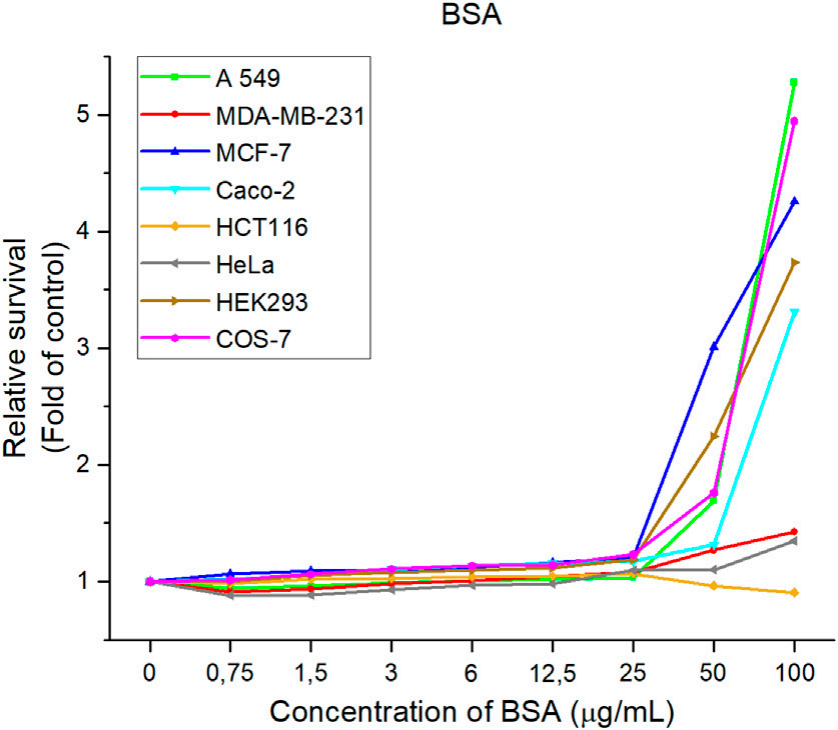
Relative cell lines survival after treatment with BSA solutions. The data are presented from three independent experiments.

**FIGURE 13:**
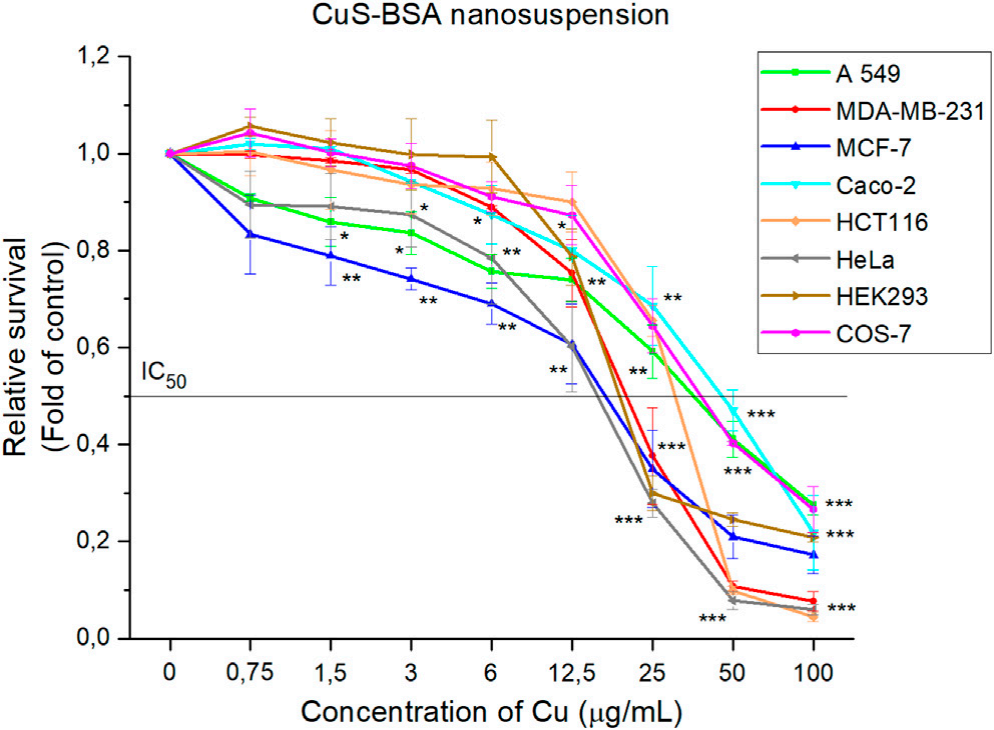
Relative cell line survival after treatment with concentration range of CuS-BSA nanosuspension. The data are presented from three independent experiments. Significantly different *p < 0.05, **p < 0.01, ***p < 0.001 vs untreated cells (control).

Changes in the metabolic activity of six cancer and two non-tumor cell lines were observed after exposure to BSA solution (Figure 12) and CuS-BSA nanosuspension (Figure 13). The obtained results showed BSA to promote cell growth and proliferation, thus alleviating the cytotoxicity of the system (Peng et al., 2013; Janani et al., 2021). A significant increasing trend was observed above the BSA concentration of 25 μg/ml in all studied lines, except HCT116 where at the maximum studied BSA concentration of 100 μg/ml a slight onset of inhibition of metabolic activity was observed (decrease by 9%). The results showed albumin as a non-toxic part of the nanosuspension supporting cell growth and proliferation in the majority of the studied lines.

On the other hand, the CuS-BSA nanocrystals show a concentration-dependent inhibition of metabolic activity on all lines studied, both tumor and non-tumor. The fastest onset of inhibition of metabolic activity appears to be as low as 0.75 μg/ml copper for the MCF-7 tumor cell line. After the application of copper concentrations above 1.5 μg/ml, cell survival began to decline rapidly in all tumor cell lines. In contrast, in non-tumor cell lines, this trend is gradual. An incipient decrease in cell survival was observed after the application of copper concentrations above 3 μg/ml for the COS-7 cell line and above 6 μg/ml for the HEK293 cell line. The most sensitive cells tested appear to be HeLa tumor cell lines (IC_50_ = 16.4 μg/ml), and MCF-7 (IC_50_ = 17.7 μg/ml), followed by the non-tumor cell line HEK293 (IC_50_ = 19.8 μg/ml) Table 1.

**TABLE 1:** IC_50_ values for six cancer and two non-tumor cell lines observed after exposure to CuS-BSA nanocrystals.

	Cancer cell lines						Normal kidney cell lines	
	HeLa	MCF-7	MDA-MB-231	HCT116	A549	Caco-2	HEK293	COS-7
IC_50_ values (Cu concentration μg/ml)	16.4	17.7	20.9	31.9	37.7	46.6	19.8	39.9

From a toxicological point of view, CuS-BSA nanosuspension could be suitable for future biomedical applications, but due to low cell selectivity, only a low dose which is non-toxic for non-tumor cell lines (copper concentration less than 3 μg/ml for the COS-7 and 6 μg/ml for the HEK293 cell lines) should be used.

Flow cytometry data (*see* Electronic Supplementary File, Supplementary Figures S2–S4) showed increase in side scatter (SSC), green (515–545 nm, FL-1), far green (>585/42 nm, FL-2) and red fluorescence (>670 nm, FL-3). Granularity changes (Supplementary Figures S2A–S4A) (increased SSC channel parameter) after CuS-BSA nanoparticles treatment displayed effective cellular uptake to the cells (HCT116, HeLa, MDA-MB-231) or affinity attachment to the cell surface. The increasing cell granularity rose in time with increasing CuS-BSA concentration dependency.

Fluorescence analysis of the NPs- containing cells (Supplementary Figures S2B–S4B) showed significant shift in FL-1, FL-2 and FL-3 fluorescence channels compared to the untreated control group. The concentrations of Cu 12.5, 25, 50 and 100 μg/ml showed the most significant shift in fluorescence intensity in tested cells (HCT116, HeLa, MDA-MB-231) compared to autofluorescence of untreated cells. These results are in accordance with granularity changes detected for the cells subjected to the same CuS-BSA concentrations. It can be concluded that during the cell culture in the presence of nanoparticles, a significant number of nanoparticles were coated onto cells, transported into the cells and stored intracellularly.

**FIGURE 14:**
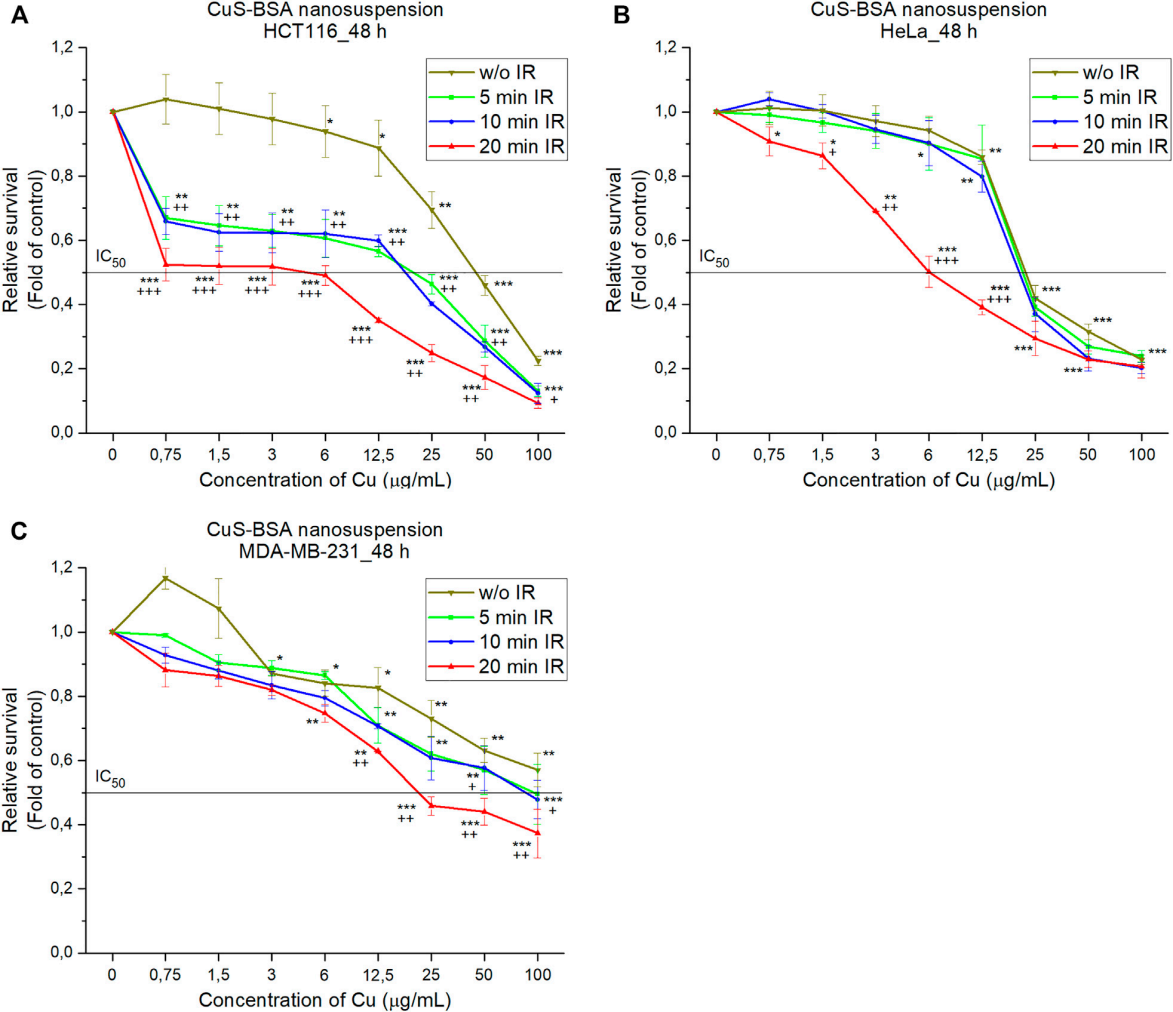
Radiation therapy of **(A)** HCT116, **(B)** HeLa and **(C)** MDA-MB-231 cells treated with CuS-BSA nanosuspensions for 48 h followed by irradiation for 5, 10 and 20 min. The data are presented from three independent experiments. Significantly different *p < 0.05, **p < 0.01, ***p < 0.001 vs untreated cells (control) and +*p* < 0.05, ++*p* < 0.01, +++*p* < 0.001 irradiated vs non-irradiated groups.

It is generally known that CuS-BSA nanoparticles have a good absorption effect in the NIR window, thus being a good candidates for photothermal ablation therapy (Chu et al., 2018). To test the suitability of the prepared CuS-BSA nanosuspension for this application, HCT116, HeLa, MDA-MB-231 cell lines containing our CuS-BSA nanocrystals were irradiated with near-infrared radiation for 5, 10 and 20 min to determine the viability of these cells compared to the untreated control. It can be seen from Figures 14A–C that as the concentration of CuS-BSA nanocrystals increases, cell viability gradually decreases. The best results were observed for the HCT116 cell line compared to the control, where the dose (IC_50_ = 25 μg/ml) ensured efficient killing of more than half of the cells. For HeLa (Figure 14B) and MDA-MB-231 (Figure 14C) cell lines, the concentration of nanocrystals does not appear to have a large effect compared to the untreated sample, but rather the irradiation dose. For the HCT116 cell line (Figure 14A), a significant decrease in viability is observed as early as 5 min of irradiation, while for the HeLa and MDA-MB-231 cell lines, a significant decrease is observed at 20 min of exposure. Photothermal destruction was induced in an irradiation dose-as well as nanoparticle concentration-dependent manner. The CuS-BSA system has been investigated several times as a promising candidate for photothermal therapy (PTT) of cancer (Chu et al., 2018; Wan et al., 2019). The CuS-BSA nanocrystals prepared by us are among these suitable candidates for PTT.

## Conclusion

We have found that the mechanochemically synthesized CuS nanoparticles prepared in an eco-friendly way are stable under human conditions. Quantitative determination of copper leaching of the CuS NPs in most simulated body fluids revealed zero copper concentration in the leachates, except simulated lung fluid (SLF, 0.015%) and simulated gastric fluid (SGF, 0.078%). The CuS-BSA nanosuspension was prepared for the first time in a simple ecological way using a two-step mechanochemical approach. Biofunctionalization of nanoparticles with albumin was performed in the second step by wet stirred media milling in 45 min. The particle size distribution showed a unimodal distribution with the average hydrodynamic diameter of 75 nm stable for at least 10 months. The TEM analysis confirmed that the sample consisted of 75 nm agglomerates, in which nanocrystalline particles with size below 20 nm are surrounded by an amorphous organic matrix. The fluorescence properties of the nanosuspension were confirmed. The UV-Vis and fluorescence quenching spectra for nanosuspension revealed that the microenvironment around the amino acid residues in BSA does not change during complex formation between BSA and nanoparticles. They have also shown the fact that increasing the concentration of CuS NPs leads to a significant decrease in fluorescence intensity as well as the proximity of tryptophan to the BSA binding site. In addition, *in vitro* experiments showed the concentration-dependent ability of CuS-BSA nanocrystals to inhibit metabolic activity on all studied cell lines, both tumor and non-tumor. On the contrary, albumin (BSA) itself has a beneficial effect on cell growth and proliferation. For biological applications, the non-toxic dose was recommended for non-tumor cell lines (copper concentration less than 3 μg/ml for COS-7 and 6 μg/ml HEK293). Moreover, the cellular uptake of CuS-BSA nanoparticles was successfully confirmed. The nanocrystals showed an efficiency to kill tumor cells (HCT116, HeLa, MDA-MB-231) upon irradiation in the NIR region in the laser dose-, as well as nanoparticle concentration-dependent manner. Our nanosuspension is a suitable candidate for photothermal ablation of cancer cells, so it is recommended for *in vivo* studies.

## Data Availability

The raw data supporting the conclusions of this article will be made available by the authors, without undue reservation.
