# Supplementary material for: Mechanochemical Preparation, Characterization and Biological Activity of Stable CuS Nanosuspension Capped by Bovine Serum Albumin
Source: Front Chem. 2022 Feb 15;10:836795. doi: 10.3389/fchem.2022.836795 (PMC8886246; doi:10.3389/fchem.2022.836795)
Supplement: Supplementary file 1 [file DataSheet1.docx]

Electronic Supplementary Information

for the paper

Mechanochemical preparation, characterization and biological activity of stable CuS nanosuspension capped by Bovine Serum Albumin

Martin Stahorský^1,2*^, Zdenka Lukáčová Bujňáková^1^, Erika Dutková^1^, Martin Kello^3^, Bogdan Mahlovanyi^4, 5^, Yaroslav Shpotyuk^4, 5^, Nina Daneu^6^, Jelena Trajić^7^, Matej Baláž^1*^

^1^ Department of Mechanochemistry, Institute of Geotechnics, Slovak Academy of Sciences, Košice, Slovakia

^2^ Faculty of Materials, Metallurgy and Recycling, Technical University of Košice, Košice, Slovakia

^3^ Department of Pharmacology, Faculty of Medicine, P.J. Safarik University, Košice, Slovakia

^4^ Institute of Physics, University of Rzeszow, Rzeszów, Poland

^5^ Department of sensor and semiconductor electronics, Ivan Franko National University of Lviv, Lviv, Ukraine

^6^ Jožef Stefan Institute, Advanced Materials Department, Ljubljana, Slovenia

^7^ Institute of Physics, University of Belgrade, Belgrade, Serbia

*** Correspondence:**Corresponding Authors
stahorsky@saske.sk
balazm@saske.sk

Fig. S1 Particle size distribution during wet stirred media milling of CuS in BSA solution determined by photon cross-correlation spectroscopy in experiments no. 1* and 2. Milling time and speed, revolutions of centrifugation and average hydrodynamic diameter (d_50_) are defined in the figure. *Nanosuspension from Experiment No. 1 was used for further investigation.


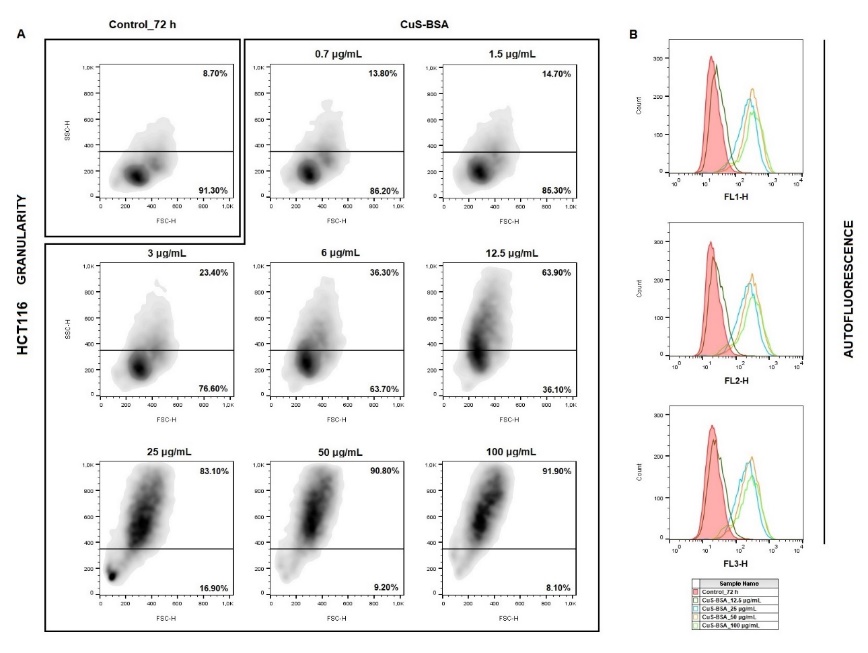


Fig. S2 Flow cytometry analyses of HCT116 cells after CuS-BSA nanosuspension treatment. (A) Granularity changes and (B) Autofluorescence analyses after 72 h exposure to range of Cu concentrations.


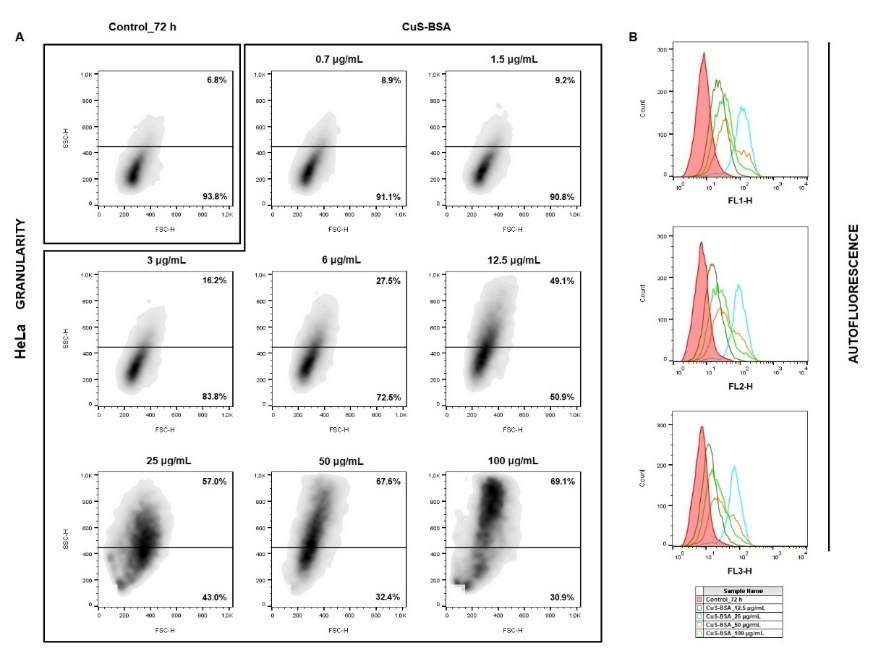


Fig. S3 Flow cytometry analyses of HeLa cells after CuS-BSA nanosuspension treatment. (A) Granularity changes and (B) Autofluorescence analyses after 72 h exposure to range of Cu concentrations.


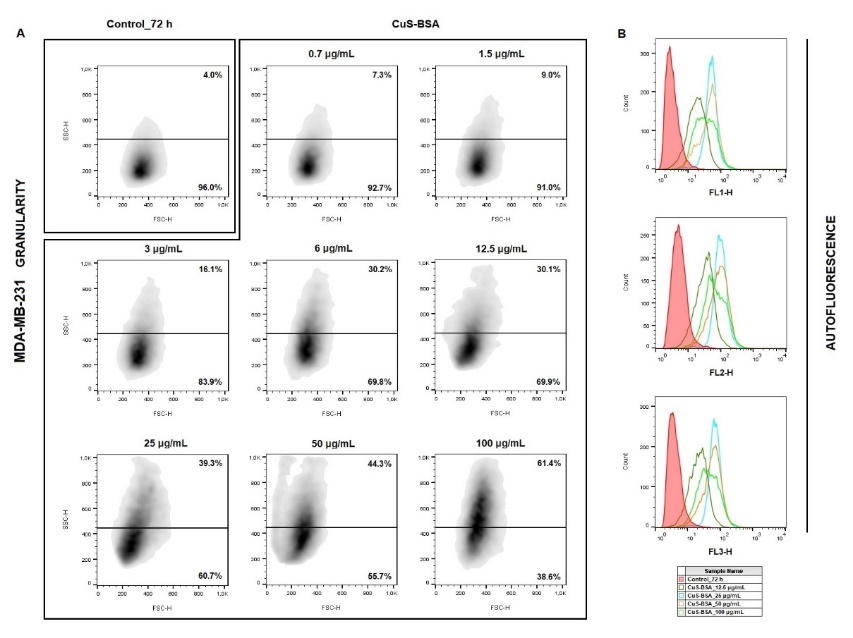


Fig. S4 Flow cytometry analyses of MDA-MB-231 cells after CuS-BSA nanosuspension treatment. (A) Granularity changes and (B) Autofluorescence analyses after 72 h exposure to range of Cu concentrations.
